# Optic nerve head: A gatekeeper for vitreous infectious insults?

**DOI:** 10.3389/fimmu.2022.987771

**Published:** 2022-09-20

**Authors:** Wenwen Lai, Jie Huang, Wangyi Fang, Saiyue Deng, Yi Xie, Wei Wang, Tong Qiao, Gezhi Xu, Xiaowei Wang, Fengfei Ding

**Affiliations:** ^1^ Department of Pharmacology, School of Basic Medical Sciences; Shanghai Key Laboratory of Visual Impairment and Restoration, Science and Technology Commission of Shanghai Municipality, Fudan University, Shanghai, China; ^2^ Department of Neurology, Tongji Hospital, Tongji Medical College, Huazhong University of Science and Technology, Wuhan, China; ^3^ Department of Ophthalmology, Eye, Ear, Nose and Throat Hospital; State Key Laboratory of Medical Neurobiology and Ministry of Education Frontiers Center for Brain Science, Institutes of Brain Science, Fudan University, Shanghai, China; ^4^ Department of Ophthalmology, Shanghai Children’s Hospital, Shanghai Jiao Tong University, Shanghai, China; ^5^ Department of Ophthalmology, University of California San Francisco, San Francisco, CA, United States

**Keywords:** optic nerve head, glial lamina cribrosa, demyelination, neuroinflammation, lipopolysaccharides, astrocyte

## Abstract

The axons of retinal ganglion cells (RGCs) pass through the optic nerve head (ONH) and form the optic nerve (ON). The ONH serves as an anatomical interface between the vitreous cavity and subarachnoid space. After inducing acute neuroinflammation by intravitreal injection of lipopolysaccharides (LPS), we observed inflammatory activation in the retina, but detect no signs of inflammation in the posterior ON or infiltration of inflammatory cells in the ONH. Therefore, we hypothesized that the ONH functions as a barrier to vitreous inflammation. Using transmission electron microscopy, we identified significant increase in G-ratio in the posterior ON on day 7 post intravitreal injection (PII) of LPS compared with the phosphate buffered saline (PBS) group. Moreover, using confocal imaging of *ex vivo* tissue extracted from *Aldh1L*1-eGFP reporter mice, we observed that the ONH astrocytes altered their spatial orientation by elongating their morphology along the axonal axis of RGCs in LPS- versus PBS-treated eyes; this was quantified by the ratio of longitudinal (D_L_) and transverse (D_T_) diameter of astrocytes and the proportion of longitudinally locating astrocytes. Supportive evidences were further provided by transmission electron microscopic imaging in rat ONH. We further conducted RNA sequencing of ONH on day 1 PII and found LPS induced clear upregulation of immune and inflammatory pathways. Furthermore, gene set enrichment analysis revealed that astrocyte and microglia contributed prominently to the transcriptomic alterations in ONH. Here, we report that the vitreous infectious insults induce morphological changes of ONH astrocytes and transcriptomic alterations in the ONH. Glial responses in the ONH may defend against vitreous infectious insults and serve as a barrier to inflammation for the central nervous system.

## Introduction

The vitreous cavity is a space that spans the distance from the posterior pole of the lens to the inner aspect of the retina, and is maintained aseptic under a physiological state. Ocular trauma or ophthalmologic operations, such as intravitreal injection and implantation of intraocular lens, could induce intraocular infection (1–3). In animal models, intravitreal injection of lipopolysaccharide (LPS) is a widely used model to study the pathophysiological mechanisms of vitreous and retinal neuroinflammation (4–7). LPS is strong inducer of glial activation, which leads to the release of pro-inflammatory cytokines in both human and animal models (8–10). In this study, we sought to establish whether vitreous infectious insults could penetrate into the posterior optic nerve (ON) that locates inside the central nervous system.

To address this scientific question, we investigated the conjunctional part between the vitreous cavity and the posterior ON, that is, the optic nerve head (ONH). The ONH shares distinctive anatomical features with the connecting parts and is composed of the lamina cribrosa (or glial lamina in rodents) and extracellular matrix (11). Inside the ONH, unmyelinated axons of retinal ganglion cells (RGCs) pass through the lamina cribrosa (or glial lamina), and share an interface with ONH fibrous astrocytes (12–16). Under normal conditions, ONH astrocytes have a flat shape with a longitudinal axis perpendicular to the RGC axons (17). In the current study, we would like to establish whether ONH astrocytes respond to vitreous LPS insults.

We first tested the infiltration of vitreous inflammation into the posterior ON through the optic nerve head (ONH). We calculated the G-ratio in axons of the posterior ON in transmission electron microscopic (TEM) images. Using both confocal imaging and TEM imaging, we characterized the morphological transformation of ONH astrocytes on day 1 post intravitreal injection (PII). We also performed RNA sequencing in ONH on day 1 PII to find evidence of neuroinflammation activation. Our results will help to integrate the functional role of the ONH in vitreous infectious insults.

## Materials and methods

### Animals

Adult Sprague-Dawley (SD) rats (220–250 g, 8-10 weeks old) were obtained from the Hubei Research Center for Laboratory Animals, Hubei, China. Transgenic *Aldh1L1*-eGFP mice (MMRRC, stock 001015-UCD) (18) (male, 8-10 weeks old) were a kind gift from Dr. Song Qin (Fudan University, Shanghai, China). The *Aldh1L1*-eGFP mice were bred on the C57BL/6J background. All rodents were housed under a 12-hour light/dark cycle with ad libitum access to food and water in a controlled animal facility. All experimental procedures and protocols were approved by the Institutional Animal Care and Use Committee of Tongji Hospital, Tongji Medical College, Huazhong University of Science and Technology, and Shanghai Medical College, Fudan University. All experiments were designed to minimize the number of animals used and animal suffering.

### Intravitreal injection of LPS

Anesthesia in rats was achieved by intraperitoneal injection of sodium pentobarbital (50 mg/kg). Retinal inflammation was induced by a single intravitreal injection of LPS (1 mg/mL, 2 μL; Sigma-Aldrich, St. Louis, MO, USA) using a 32G Hamilton microinjector (Hamilton Co., Reno, NV, USA) under a dissecting microscope. LPS was hand injected slowly over 30 s and sterile phosphate-buffered saline (PBS; 2 μL) was administered to the contralateral vitreous cavity in the same manner immediately after LPS administration as the vehicle control. To characterize the morphological changes in ONH astrocytes, the *Aldh1l1*-eGFP reporter mice were administered the same injections but with a 1μL injection volume for both LPS and PBS.

### Tissue sample preparation

On day 1 or day 7 PII, the rats were intracardially perfused with PBS (pH 7.4) followed by 4% paraformaldehyde (PFA) while being fully anesthetized by a ketamine (80mg/kg) and xylazine (10mg/kg) mixture. The eye with the ON was enucleated and post-fixed in 4% PFA at 4 °C overnight. Next, the tissue samples were dehydrated in a sucrose gradient (10%,20%, and 30% for12 hrs each). For immunofluorescence detection of molecular targets in the posterior ON, the ON was embedded in an optimal cutting temperature (OCT) compound, and then sectioned into 10 μm thick transverse sections, taken from 1 mm behind the eye. Approximately the posterior ON within 1-1.5 mm behind the optic cup (proximal segment) were used for immunostaining or transmission electron microscopy. To study the changes in the ONH, the eye cup with the initial 1 mm optic nerve was embedded in OCT and cut into 10 μm thick sagittal sections and only sections containing the ONH zone were used. To study LPS-induced changes in the retina, the eye cup was embedded in OCT and cut into 10 μm thick sagittal sections after removing the cornea and lenses. A Leica cryostat (Leica CM1950, Wetzlar, Germany) was used for all thin sectioning. Following sectioning, samples were stored at -80 °C until use.

### Immunofluorescence imaging

Immunofluorescence staining was conducted in the tissue sections generated from the fixed retina, ONH, or posterior ON tissue samples on day 1 or day 7 PII of LPS or PBS. After washing twice with PBS, the sections were immersed in PBS containing 10% bovine serum albumin (Servicebio, GC305010-25G, China) and 0.3% Triton X100 (Servicebio, GC204003, China) for 60 min and then washed for three times with PBS. Subsequently, the sections were incubated with one or more of the following primary antibodies diluted in PBS containing 5% bovine serum albumin and 0.3% Triton X100: anti-glial fibrillary acidic protein (GFAP) antibody (1:500, mouse; Invitrogen, Carlsbad, CA, USA), anti-ionized calcium binding adaptor molecule 1 (IBA1) antibody (1:500, rabbit; Wako Pure Chemical, Osaka, Japan), anti-myelin basic protein (MBP) antibody (1:200, rat; Millipore, Bedford, MA, USA), anti-CD68 (1:200, mouse anti-rat; Biorad, Hercules, CA, USA) and anti-Aquaporin 4 (AQP4) antibody (1:500, rabbit; Protein Tech, China) at 4 °C overnight. After washing with PBS, the sections were incubated with the fluorescent secondary antibody: goat anti-mouse-Alexa Fluor 488 (1:500; Invitrogen), goat-anti-rabbit-Cyanine 3 (Cy3, 1:500, Invitrogen), or donkey-anti-rat Alexa Fluor 488 (1:500; Invitrogen) for 1 hr at room temperature. Excess secondary antibodies were washed off with PBS and DAPI containing mounting medium (Beyotime, Beijing, China) was applied. Images of the retina and ON were captured at 10 to 40X magnification by an epifluorescence microscope (Olympus, Tokyo, Japan, DP72). Three sections per retina, five sections per ON, three sections per ONH were analyzed by ImageJ software (National Institutes of Health, Bethesda, MD, USA). To quantify the thickness of retina, six non-overlapping image field at 20 X magnification were taken to calculate position-specific thickness. All settings were consistent between the control and experimental groups for comparison of fluorescence intensity.

### Hematoxylin-eosin staining

For sagittal sections extracted from the eyes on day 1 PII of LPS or PBS, hematoxylin-eosin (HE)staining was conducted according to the manufacturer’s instructions. Briefly, sections were stained with hematoxylin solution (Beyotime, C0105S) for 5 min followed by 5 dips in 1% acid ethanol (1% HCl in 70% ethanol) and then rinsed in distilled water for 5 min. The sections were stained with eosin solution (Beyotime) for 3 min before rinsing in distilled water for 5min, followed by dehydration with a gradient solution of ethanol (70% ethanol for 10 s, 80% ethanol for 10 s, 90% ethanol for 10 s, and absolute ethanol for 10 s) and then submersion in xylene. The mounted slides were examined and photographed using a light microscope (Olympus) at 10X magnification. The inflammatory cells infiltrating into the retina, vitreous cavity and ONH were evaluated.

### Confocal imaging of ONH astrocyte morphology

On day 1 PII of LPS in one eye and PBS in the other eye of the *Aldh1l1*-eGFP mice, bilateral ONs along with the retina were carefully dissected and post fixed with 4% PFA at 4 °C over night. The entire ON was placed on a glass slide and sealed in Fluoromount-G^®^ (Southern Biotech, Birmingham, AL, USA). High-resolution images (1024 × 1024 pixels) were captured in the ONH area using a confocal microscope (Leica TCS SP8, Germany) at 40X magnification. Z-stacks of the ONH were taken at 1μm step for three-dimensional (3D)-reconstruction. Representative images for ONH astrocyte morphology were created as max projections using ImageJ software.

### Imaging by fluorescence micro-optical sectioning tomography imaging system (fMOST)

On day 1 PII of LPS in one eye and PBS in the contralateral eye of the *Aldh1l1*-eGFP mice, bilateral ONs along with the retina till the optic chiasma were carefully dissected and post fixed with 4% PFA. After dehydration with gradient alcohol solutions (25%, 50%, 75%, 100%), the tissue was embedded in Lowicry HM20 resin (Warrington, PA, USA) before imaging using an fMOST system (BioMapping5000, Wuhan OE-Bio Co., Ltd., Hubei, China) as described previously (19). Briefly, the diffraction-limited illumination line was provided by an optical system, which included a cylindrical lens, a tube lens, and a high NA water objective lens (LUMPLFLN 40X water, NA 0.8, Olympus). The fluorescent signals were captured by a scientific complementary metaloxide semiconductor (sCMOS) camera (ORCA-Flash 4.0, Hamamatsu Photonic K. K., Shizuoka, Japan) with high sensitivity and speed. The sample was fixed on a precision motion stage (Aerotech, Pittsburg, PA, USA), possessing high repeatability (± 0.2 μm) and stability. A tissue layer of 1-μm thickness was removed with a diamond knife after completing the imaging of the top layer in the coronal plane. The cycle of imaging and sample sectioning was continuously conducted until data acquisition was completed. All captured images were automatically registered, reconstructed, and stored for data processing resolution: 0.35 μm x 0.35 μm x 2 μm.

### Transmission electron microscopic imaging

TEM imaging was performed in the posterior ON (between 1-1.5 mm) behind the optic cup for measurements of G-ratio. On day 7 PII, rats were intracardially perfused with 4% PFA containing 2.5% glutaraldehyde while being fully anesthetized using a ketamine(80mg/kg) and xylazine(10mg/kg) mixture. Tissues were then post fixed with 1% osmium tetroxide, in 0.1 M PB for 2 hrs at room temperature avoiding light. After removing osmium tetroxide, the tissues were rinsed in 0.1 M phosphate buffer (PB) for three times. Tissues were sequentially dehydrated in 30%, 50%, 70%, 80%, 95%, and 100% alcohol for 20 min in each solution and 100% acetone twice for 15 min for each. After resin penetration and embedding, the samples were polymerized in a 65 °C oven for at least 48 hrs. The resin blocks were cut to 60-80 nm thickness on an ultra-microtome (Leica UC7), and the tissues were fished out onto the 150 meshes cuprum grids with formvar film. The samples were immersed in 2% uranium acetate saturated alcohol solution to stain for 8 min avoiding light, subsequently rinsed in 70% ethanol for three times and another three times with distilled water. After staining with 2.6% lead citrate for 8 min, the sample was rinsed with distilled water for three times. Samples were processed for transmission electron microscopy (HT7800, Hitachi, Tokyo, Japan) at 80 kV. At 6000 × magnification, the length of the inner diameters (axon) and outer diameters (axon and myelin sheath) of all axons was measured using ImageJ software. The G-ratio was calculated as the ratio of inner to outer diameters. In separate experiments, transmission electron microscopic imaging was performed in the ONH on day 1 PII of LPS and PBS with transmission electron microscope (Philips, CM120) at 120 kV. The optic nerve connecting with partial retina was embedded. Coronal sections with thickness of 50-60 nm were taken within 200 μm behind the central zone. The image features of the ONH was identified by bundles of unmyelinated axons embraced. The morphological features astrocyte nucleus and cellular processes were identified based on the previous literatures and checked by experienced histologists (20).

### RNA extraction and quantitative Real-time polymerase chain reaction

To extract the total RNA of the retina and posterior ON on day 1 PII, anesthetized rats were intracardially perfused with PBS. The ON at 1 mm from the eye and retina was isolated and the sclera and meningeal sheath were carefully removed under a dissecting microscope. Total RNA was extracted from the retina and ON using the TRIzol RNA reagent (Invitrogen), and cDNA was synthesized using PrimeScript™ RT Master Mix (Perfect Real Time) (TaKaRa, Shiga, Japan) according to the manufacturer’s instructions. Quantitative polymerase chain reaction (PCR) was performed using Hieff qPCR SYBR Green Master Mix (No ROX) according to the manufacturer’s instructions (YEASEN, Shanghai, China). The expression level of each mRNA was normalized to that of *Gapdh* mRNA and calculated using the 2^−ΔΔCt^ method. All primer sequences are provided in **Supplementary Table 1**.

### RNA sequencing and bioinformatic analysis

On day 1 PII, enucleated globes were rapidly submerged in cold PBS, and the anterior segment, lens, and retina were removed. Using a trephine, the translucent unmyelinated ONH tissue was carefully dissected under a dissecting microscope. A single ONH was used as a biological replicate for the following procedures. Total RNA was extracted from the tissue using TRIzol (Invitrogen) according to the manufacturer’s instructions and genomic DNA was removed using DNase I (TaKara). The RNA quality was determined by 2100 Bioanalyser (Agilent) and quantified using the ND-2000 (NanoDrop Technologies, Wilmington, DE, USA). The RNA-seq transcriptome library was prepared following Clontech-SMART-Seq v4 Ultra Low Input RNA Kit for Sequencing using 10 ng of total RNA. Subsequently, the synthesized cDNA was conducted into the library according to the Illumina library construction protocol (San Diego, CA, USA). Finally, the paired-end RNA-seq sequencing library was sequenced with an Illumina NovaSeq 6000 sequencer (2 × 150 bp read length) and the data were analyzed on the Majorbio Cloud Platform (http://www.majorbio.com). To identify differentially expressed genes (DEGs) between the two groups, the expression level of each transcript was calculated as the transcripts per million reads (TPM). Differential expression analysis was performed using the statistical DESeq2 (p-adjust ≤0.01and | log2Fold Change | ≥ 1). Additionally, functional annotation analysis including Gene Ontology (GO) and Kyoto Encyclopedia of Genes and Genomes (KEGG) were performed to identify which DEGs were significantly enriched in GO terms and metabolic pathways at Bonferroni-corrected P-adjust value ≤0.01. One sample from each group was excluded as the outliners according to principal component analysis (PCA), which showed a striking difference from the other samples.

### GSEA based on the reference-based cell type-specific gene sets

Gene set enrichment analysis was performed on the Majorbio Cloud Platform (http://www.majorbio.com). The cell type-specific gene sets for astrocyte, microglia, oligodendrocyte and neuron were summarized as described previously (**Supplementary Table 2**) (21–23). Differential expression analysis was performed using the statistical DESeq2 (p-adjust ≤0.05 and |log2Fold Change| ≥ 1). Normalized enrichment scores (NES) were used to measure the magnitude of enrichment. The leading-edge genes in GSEA are the genes that most strongly contribute to the detected enrichment signal of a pathway (24), which were listed with their respective enrichment scores (ES). The enrichment plot was output from the analytical platform for each cell type.

### Measurements of the retinal thickness in tissue sections

Retina thickness was measured in sections cutting through the center of the optic nerve head. The tissue samples were prepared from the retina on day 1 and 7 PII of PBS and LPS. DAPI staining and GFAP signal were used to measure the retinal thickness from ganglion cell layer to outer nuclear layer using ImageJ. Six images of 20 x magnification were taken at position 1-6 on the retina (illustrated in **Supplementary Figure 1**). The positions were kept consistent across all samples. Interval equivalent to two image field was skipped before taking the next representative image. Three repetitive measurements were performed and averaged for each image. The symmetric positions (1 and 6, 4 and 5, 3 and 4 in **Supplementary Figure 1**) were grouped together for comparisons between the PBS and LPS groups.

### Quantification of the ratio of transverse and vertical diameters of ONH astrocytes

Confocal images of *Aldh1L1*-eGFP mice were used to analyze the ratio of transverse (D_T_)and longitudinal (D_L_) dimensions of ONH astrocytes using image J. The directions of the astrocyte were defined relative to the ON. The distance between the farthest processes in the transverse or longitudinal directions was measured as D_T_ or D_L_ respectively. Astrocytes where D_L_ was longer than D_T_ were defined as longitudinally oriented astrocyte. The ratio of D_L_/D_T_ and the number of longitudinally oriented (D_L_>D_T_) astrocytes were quantified.

### Sholl analysis of microglia in the ONH and ON

For both ONH and posterior ON samples, 15-20 microglial cells from three to five images were quantified for each sample. Seven ONHs per group and six posterior ONs were used for statistical analysis. Sholl analysis was performed as previously described with slight modification (25). Each image was thresholded and analyzed with the Sholl analysis plugin of ImageJ software. We set the first shell at 5 μm and subsequent shells in 1-μm steps to determine the number of intersections at each Sholl radius.

### Three-dimensional (3D) construction of astrocyte morphology

Three-dimensional construction of ONH astrocyte morphology was performed using Imaris 9.8 (Bitplane, South Windsor, CT, USA) based on the imaging data generated from confocal imaging and fMOST. Image stacks covering the target cell were selected and imported into imaris. Following the seed-based standardized protocol, the surface covering the soma and cell processes were established in Imaris. Manual modification of the established surfaces ensured the accuracy of cell boundaries, while the surfaces were color-coded for demonstration.

### Statistical analysis

All data are presented as the mean ± standard derivation (SD). Statistical analyses were conducted in GraphPad (Prism Version 8.0.1). The statistical differences between groups were measured using unpaired t test, Mann-Whitney test or two-way analysis of variance (ANOVA). P-values < 0.05 were considered statistically significant.

## Results

### Intravitreal LPS injection induces obvious neuroinflammation in the retina but not in the posterior ON

**Figure 1:**
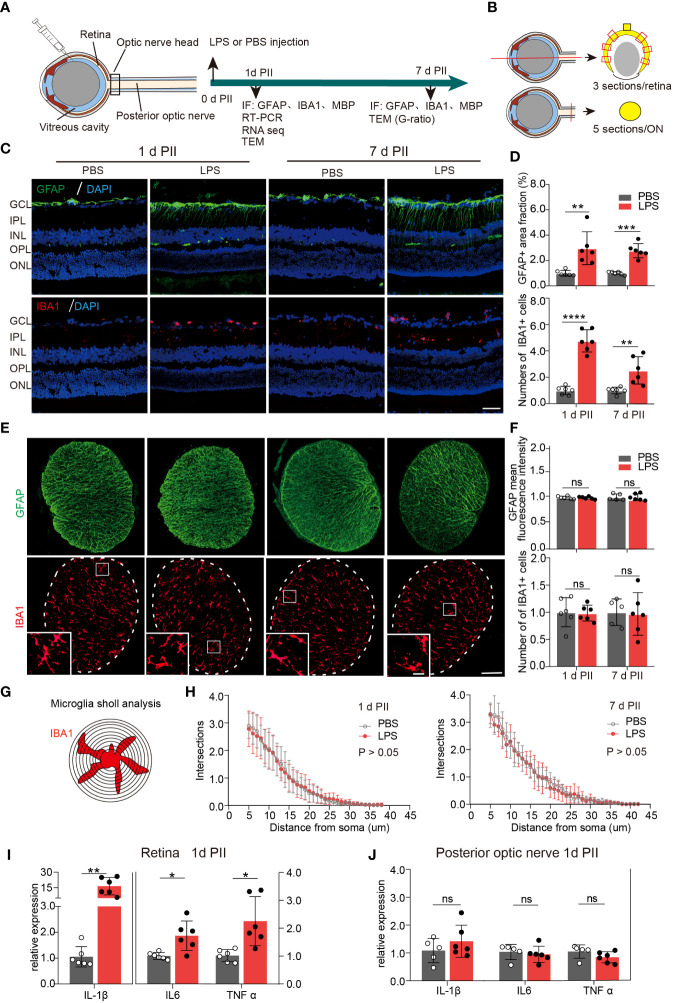
Intravitreal LPS injection induces obvious neuroinflammation in the retina but not in posterior ON. **(A)** Diagram of the experimental design. **(B)** Schematic illustration of the locations and orientations of extracting sections for immunostaining in the retina (upper panel) and posterior ON (lower panel). **(C)** Representative images of immunofluorescence staining of GFAP (green), IBA1 (red) and DAPI (blue) in the retina on day 1 and 7 post PBS or LPS intravitreal injection (1d PII or 7d PII). GCL, Ganglion cell layer; IPL, Inner plexiform layer; INL, Inner nuclear layer; OPL, Outer plexiform layer; ONL, Outer nuclear layer. Scale bar, 50 μm. **(D)** Histograms summarizing the fraction of the GFAP-positive staining area and the number of IBA1 positive cells in the immunofluorescence staining demonstrated in panel C; n = 6. **(E)** Representative images of immunofluorescence staining of GFAP (green) and IBA1 (red) in posterior ON on day 1 and 7 PII of PBS or LPS. Scale bar, 100 μm for coronal sections of the ON, 20 μm for inserts (lower panel). **(F)** Histograms summarizing the mean fluorescence intensity of GFAP and the number of IBA1-positive cells in the immunofluorescence staining demonstrated in panel E; n = 5-6. **(G)** Schematic illustration of microglia Sholl analysis in the coronal sections of the posterior ON; **(H)** Sholl plot demonstrating the number of intersections between the microglia branches and each increasing concentric circle plotted against the distance away from the soma (μm) based on IBA1 staining in the ON on day 1 (left panel) or day 7 (right panel) PII of PBS or LPS demonstrated in panel E; n =6. **(I, J)** mRNA expression levels of IL-1β, IL-6, and TNF-α in the retina and ON on day 1 PII. The expression levels of the LPS group were normalized to those of the PBS group; n = 5-6 per group. All data are presented as the mean ± SD. Unpaired t-test or Mann-Whitney test was performed for statistical analysis or two-way repeated measures ANOVA was performed for statistical analysis; n.s., P > 0.05, *P < 0.05, **P < 0.01, ***P < 0.001, ****P < 0.0001., ns, non-significant.

On day 1 and 7 PII of LPS, we stained the sections of the retina and ON for glial fibrillary acidic protein (GFAP, astrocyte marker) and ionized calcium binding adaptor molecule 1 (IBA1 microglial marker) (**Figures 1A**, **B**). GFAP staining labeled the fibrous structure radiating from ganglion cell layers (**Figure 1C**), which was consistent with the observations of previous studies (26, 27).The GFAP-positive area was significantly increased in LPS-treated retinas compared with the PBS-treated retinas at both time points (**Figure 1D**). In contrast, GFAP staining in the ONs was comparable between the PBS and LPS-treated eyes (**Figure 1F**). IBA1 staining labeled the cells distributed throughout all layers of retina, mainly in the ganglion cell layer (GCL) and the inner plexiform layer (IPL) (**Figure 1C**). IBA1 positive cell numbers were significantly increased by approximately 5-fold (PII 1) and 2.5-fold (PII 7) in the LPS-treated retina compared with the PBS-treated retina (**Figure 1D**), but no significant changes were found in the ONs between the two groups (**Figure 1E**). Additionally, Sholl analysis showed that the morphological features of the ON microglia remained unaltered on day 1 and 7 PII (**Figures 1G, H**). Retinal thickness measured in histology sections were comparable between the PBS and LPS groups on both time points (**Supplementary Figure 1**). Using RT-PCR, the mRNA levels of IL-1β, TNF-α, and IL-6 were significantly elevated in retinas treated with LPS on day 1 PII, with a relative increase ranging from 1.5 to 15-fold compared with the PBS-treated retinas (**Figure 1I**). However, no significant changes in these genes were found in the posterior ONs generated on day 1 PII of PBS or LPS (**Figure 1J**). In summary, intravitreal LPS injection induced significant neuroinflammation in the retina but not in the posterior ON on day 1 and 7 PII.

### Intravitreal LPS injection increases the diameter and G-ratio of axons in posterior ON

**Figure 2:**
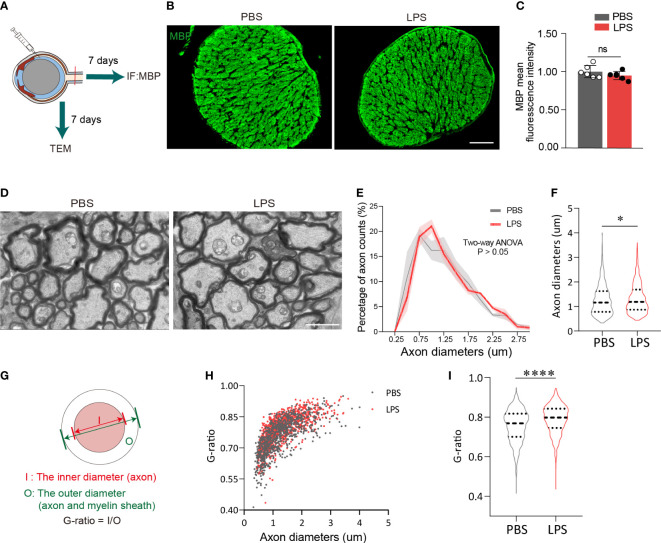
Intravitreal LPS injection increases the diameter and G-ratio of axons in posterior ON on day 7 PII. **(A)** Diagram illustrating the experiments measuring demyelination in the ON on day 7 PII. **(B)** Representative images of immunostaining of MBP in the ON. Coronal sections (upper panel), zoom-in view of the selected regions (lower panel). Scale bar, 100 μm, upper panel. **(C)** Histograms summarizing the fluorescence intensity of MBP in PBS and LPS groups on day 7 PII; n = 6 per group. Data are normalized to the average of the PBS group. **(D)** Representative images of ONs captured by transmission electrical microscopy (TEM) for the PBS and LPS groups on day 7 PII. Scale, bar, 2 μm. **(E)** Histogram displaying the distribution of axonal diameters and numbers for both groups. The axonal diameter shown in the histogram ranges from 0.25 to 3.0 μm. **(F)** Statistical comparisons of all the axonal diameters between PBS and LPS groups (n = 828 axons for the PBS group and n = 1006 axons for the LPS group; n = 3 ONs per group). **(G)** Schematic illustration of the calculation strategy for the G-ratio. **(H)** The distribution of the G-ratio plotted against axonal diameters for both groups. **(I)** Statistical comparisons of the G-ratio between PBS and LPS groups of all the axons. All data are presented as the mean ± SD. Unpaired t-test, Mann-Whitney test, or two-way ANOVA was performed for statistical analysis, n.s., P > 0.05, *P < 0.05, ****P < 0.0001.

Based on the observation of neuroinflammation in the retina after intravitreal LPS injection, we next investigated whether the axons and myelin sheath of RGCs in posterior ON were also affected. On day 7 PII, the fluorescence intensity of myelin basic protein (MBP) from both groups was evenly distributed with no significant differences between groups (**Figures 2A–C**). Using transmission electron microscopy to quantify the relative thickness of mature myelin sheets in the posterior ON (**Figure 2D**), we found that the majority of imaged axons (PBS group, 828 axons from three mice; LPS group, 1006 axons from three mice) had diameters of approximately 1 μm, and that the distributions in count numbers with different axonal diameters were comparable between the two groups (**Figure 2E**). A slight but significant increase in axonal diameters in the ONs was observed in LPS-treated eyes versus control, indicating axonal swelling (P<0.05, **Figure 2F**). To quantify LPS-dependent changes in the myelin sheath, the G-ratio was calculated as the ratio of the axonal diameter (I) to the diameter of myelinated nerve fibers (O) (**Figure 2G**), which is used to evaluate the demyelination with high sensitivity (28). The G-ratio was significantly higher in ONs of LPS-treated eye versus controls (P<0.0001, **Figures 2H, I** **)**. In summary, intravitreal LPS injection significantly increased G-ratio in axons of the posterior ON characterized by transmission electron microscopy on day 7 PII, which could be due to myelin loss and/or axonal swelling.

### Inflammatory cell infiltration does not occur in the ONH on day 1 PII

**Figure 3:**
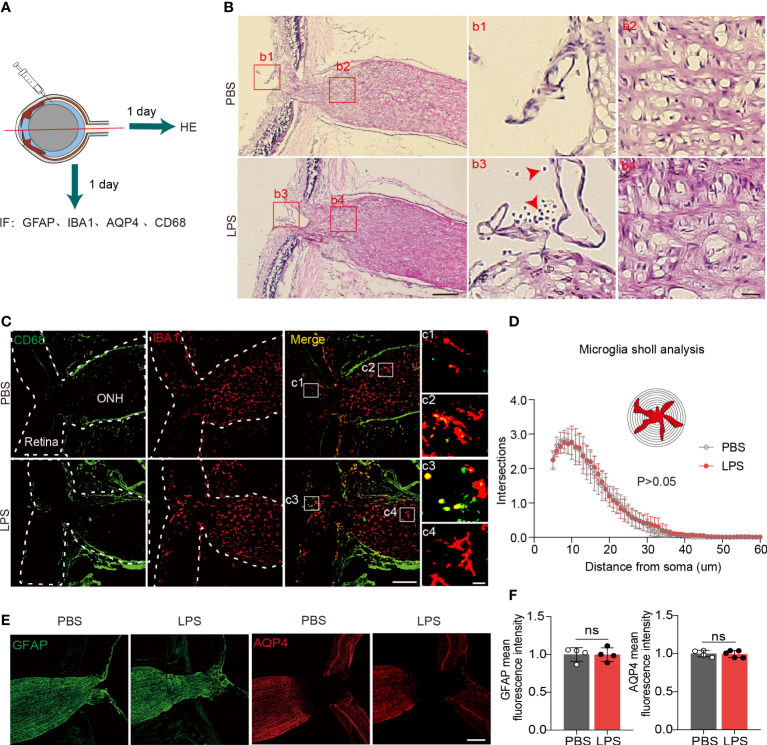
Inflammatory cell infiltration and microglial activation in the ONH are not present on day 1 PII of LPS. **(A)** Diagram illustrating the experiments measuring inflammatory cell infiltration in the ONH on day 1 PII. Abbreviations: AQP4, aquaporin 4; CD68, also called macrosialin. **(B)** Representative images of hematoxylin and eosin (H&E) staining in the sagittal sections including the retina, ONH and ON on day 1 PII of PBS or LPS. Red square boxes are zoomed and displayed on the right. Regions b1 and b3 from the vitreous cavity, and regions b2 and b4 from the area of the ONH. Red arrows indicate inflammatory cells stained by H&E. Scale bar, 200 μm for the zoom-out view, 20 μm for the inserts of b1-b4, n = 7 ONs per group. **(C)** Representative images of immunofluorescence staining for CD68 and IBA1 in sagittal sections covering the partial retina and ONH. Regions c1 and c3 from the area of the vitreous cavity, and regions c2 and c4 from the area of the ONH. Scale bar, 200 μm for the zoom-out view (left), 20 μm for the inserts of c1-c4; n = 7 ONHs per group. **(D)** Sholl plot based on IBA1 immunofluorescence staining in the ONH. n = 7 ONH per group. **(E)** Representative images of immunofluorescence staining for GFAP and AQP4 in sagittal sections covering the partial retina, ONH and partial ON for both groups. Scale bar, 100 μm. **(F)** Histograms summarizing the mean fluorescence intensity of GFAP and AQP4 for both groups. Data are normalized to the average of the PBS groups. n = 4-5 ONs per group. Unpaired t test or two-way repeated measures ANOVA was performed for statistical analysis, ns, non-significant, P > 0.05.

To evaluate the penetration of inflammatory cells from vitreous into the ONH and posterior ON, we observed HE+ cells that were present in the retinas of LPS-treated eyes but absent in PBS-treated eyes, which indicate the LPS-induced vitreous neuroinflammation (**Figures 3A, B**). In LPS-treated eyes, we found no infiltration of HE+ cells in the ONH (**Figure 3B**). In the sagittal sections showing the partial retina and ONH, CD68 + cells were present only in the retina and vitreous cavity but were absent in the ONH (**Figures 3C**, c3 and c4). Sholl analysis showed that ONH microglial morphological features remained unaltered in the ONH in LPS- and PBS-treated eyes (**Figure 3D**). Fibrous astrocytes in the rodent ONH form the glial lamina, and astrocytic processes are transversely oriented with respect to the long axis of the ON (16, 29). The astrocytic marker GFAP in sagittal sections of the ONH exhibited fluorescence intensity in both PBS- and LPS-treated eyes (**Figure 3E**). Aquaporin 4 (AQP4), water channels that are extensively expressed in protoplasmic astrocytes, was absent in the glial lamina in the ONH, which was consistent with the previously reported observation (30) (**Figure 3F**). Herein, we did not find infiltrating inflammatory cells from the LPS-treated vitreous into the ONH, and the microglial morphology and astrocytic GFAP in the ONH was unaltered on day 1 PII of LPS.

### Vitreous inflammatory insults induce astrocytic morphological changes in the ONH on day 1 PII

**Figure 4:**
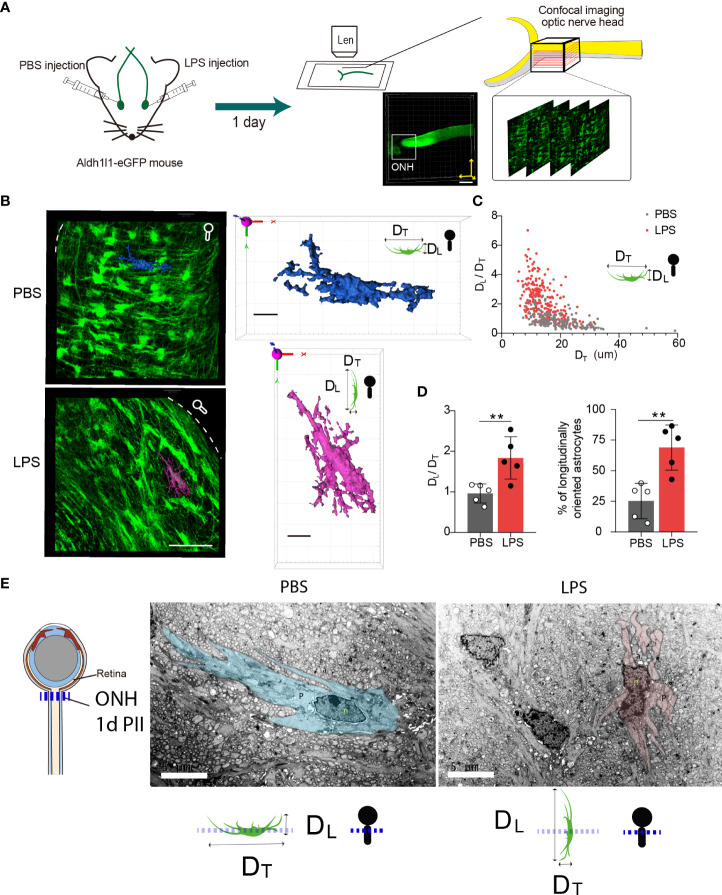
Intravitreal LPS injection triggers significant morphological transformation in the ONH astrocytes on day 1 PII. **(A)** Schematic illustration of the experiment procedure for confocal imaging in the fixed tissue sample of the ONH generated from Aldh1L1-eGFP reporter mice. **(B)** Representative images generated from z-projection of xy-z confocal imaging stacks in the ONH for both groups. Scale bar, 50 μm. Circle-rod shape in white at the upper right simulates the orientation of the retina and ON. Color-code 3D-construct cell surfaces exemplify the morphological features of ONH astrocytes in both groups; scale bar, 10 μm, DT indicates the diameter of the cell domain along the coronal axis of the ONH; DL indicates the diameter of cell domain along the longitudinal axis of the ONH. **(C)** Distribution of the ratio of DL/DT plotted against the DT (unit, μm) for both groups; n = 177 cells for the PBS group, and n =245 cells for the LPS group. **(D)** Statistical comparisons of the ratio of DT/DL, the fraction of longitudinally oriented astrocytes between the PBS and LPS groups; n = 5 ONs per group. **(E)** Transmission electron microscopic imaging of rat ONH astrocyte on day 1 PII of PBS and LPS. The representative astrocyte morphology was outlined by pseudo color (PBS, blue) and (LPS, pink). Scale bar, 5 μm. All data are presented as the mean ± SD. Unpaired t-test was performed for statistical analysis, **P < 0.01.

Using Ald1l1-eGFP reporter mice, we observed the morphological features of ONH astrocytes with confocal imaging (**Figure 4A**). We constructed the 3D surfaces using software from confocal imaging stacks to display the morphological features in the ONH of LPS- and PBS-treated eyes. The z-projection of 10-20 frames of consecutive images clearly demonstrated that the ONH astrocytes in PBS-treated eyes had the polygon-shaped cell somas and transversely exerted cell processes vertical to the orientation of axons, whereas ONH astrocytes in LPS treated eyes exhibited relatively long and narrow cell somas along the axonal orientation with rare transverse cell processes (**Figure 4B**). In the 3D-constructed surface demonstrations, we clearly identified that the ratio of transverse (D_T_) and longitudinal (D_L_) diameters defined according to the axonal orientation were flipped by PII of LPS (**Figures 4B, C**). By comparing the ipsilateral (LPS) and contralateral (PBS)-treated eyes, the ratio of D_L_/D_T_ was significantly elevated in the LPS-treated eyes compared with the PBS-treated eyes (**Figures 4C, D**). Quantification of the longitudinally oriented astrocytes (D_L_ > D_T_) in the ONH showed an evident increase in the proportion of eGFP-positive cells (**Figure 4D**). Furthermore, in a separate experiment, we performed volumetric imaging of the entire eye tissue extracted from one *Aldh1l1*-eGFP mouse using the fMOST technique (**Supplementary Figure 2**). The partial retina connected to the postocular ONs until the optic chiasma was visualized by post-imaging construction (**Figure 4F**). Coronal segmentation revealed the ONH astrocyte morphological features in the ipsilateral (LPS) and contralateral (PBS) eyes (**Supplementary Figure 2**). Although the astrocytic cellular processes were not fully illustrated by the fMOST technique, we observed that the cell territory in the coronal sections was relatively restrained in the LPS-treated eye versus the PBS-treated eye, which could be due to the altered orientation of astrocyte morphology induced by vitreous LPS insults (**Supplementary Figure 2**). In addition, we performed the transmission electron microscopic imaging in rat ONH on day 1 PII. The bundles of unmyelinated RGC axons were embraced by sparsely distributing astrocytic cellular processes. The ONH astrocyte of PBS group exhibited organized cytoplasm as well as long and continuous cellular processes in the coronal plane, whereas the ONH astrocyte of LPS group showed narrowed cytoplasm as well as short and interrupted cellular processes that represent longitudinally oriented processes being sectioned coronally (**Figure 4E**). The morphology features support the confocal imaging findings. Additionally, we observed tangling of the astrocyte processes following LPS administration (**Figure 4E**). In summary, vitreous LPS insults induce morphological transformation of ONH astrocytes on day 1 PII.

### RNA sequencing showed transcriptome changes in the ONH induced by intravitreal LPS injection

**Figure 5:**
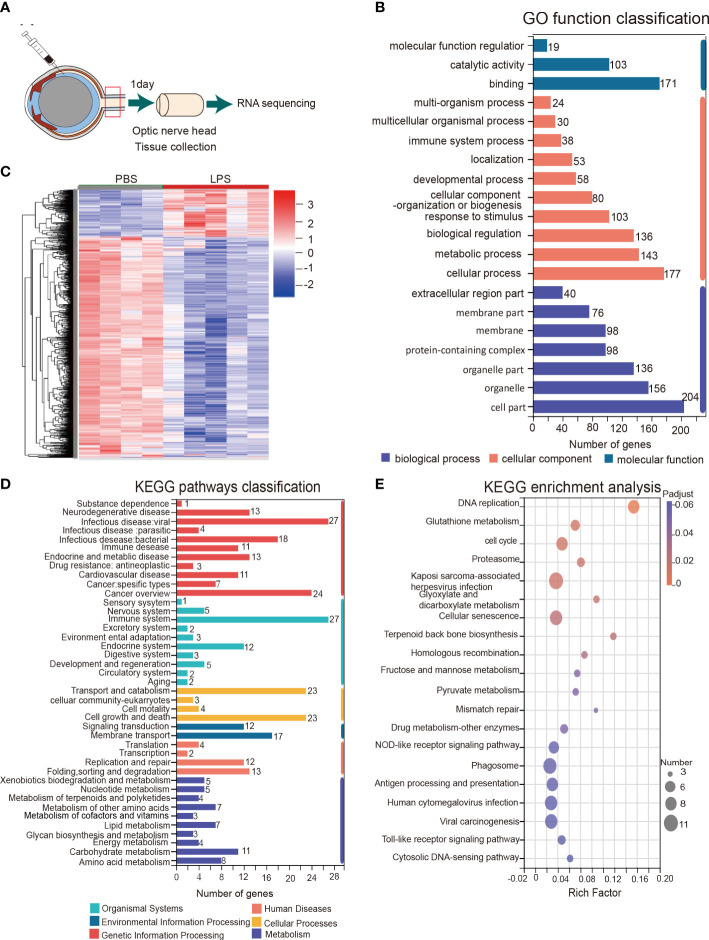
KEGG pathway and gene ontology (GO) analysis of the transcriptomes of the ONH on day 1 PII. **(A)** Schematic illustration of ONH RNA sequencing; n = 4 ONHs for the PBS group and n = 5 ONHs for the LPS group. **(B)** GO function classification of the up-regulated differentially expressed genes (DEGs) in the LPS group compared to the PBS group. P-adjust value ≤0.01. **(C)** Cluster heatmap demonstrating the transcriptomic features of DEGs in the PBS and LPS groups. Color scale indicates the normalized expression level of DEGs. **(D)** KEGG pathways classification of up-regulated DEGs. P-adjust value ≤ 0.01. **(E)** KEGG enrichment analysis based on the up-regulated DEGs.

To further understand the ongoing pathophysiological mechanisms in the ONH, we conducted RNA sequencing in fresh tissue samples extracted from rats ONH on day 1 PII (**Figure 5A**). RNA-seq revealed 1221 DEGs between the PBS and LPS-treated groups, including 1006 down-regulated genes and 215 up-regulated genes (**Supplementary Table 3**). Cluster analysis displayed a striking transcriptomic difference between LPS and PBS-treated groups (**Figure 5C**). GO analysis highlighted the GO terms with high enrichment of DEGs (>100 genes), including catalytic activity and binding (molecular function); response to stimulus, biological regulation, metabolic process, and cellular processes (biological process); organelle part, organelles, cell part (cellular component) (**Figure 5B**). The top up or down-regulated genes were displayed with respective expression levels (**Supplementary Figure 3**). KEGG pathway analysis showed that the pathways with more than 20 up-regulated genes included infectious disease: viral, cancer overview (Human disease); cell growth and death, transport and catabolism (Cellular processes); and the immune system (Organismal systems) (**Figure 5D**). The enriched KEGG pathways with statistical significance are listed, including phagosome, antigen processing and presentation, Toll-like receptor signaling pathway, cellular senescence, proteasome, cell cycle, and glutathione metabolism (**Figure 5E**). In summary, RNA sequencing of the ONH revealed striking transcriptomic alterations induced by vitreous LPS insults compared with PBS as well as dominantly upregulated pathways involved in infection, immune reactions, and inflammation.

### Astrocytic and microglial cell type-specific enrichment were identified in the ONH transcriptome

**Figure 6:**
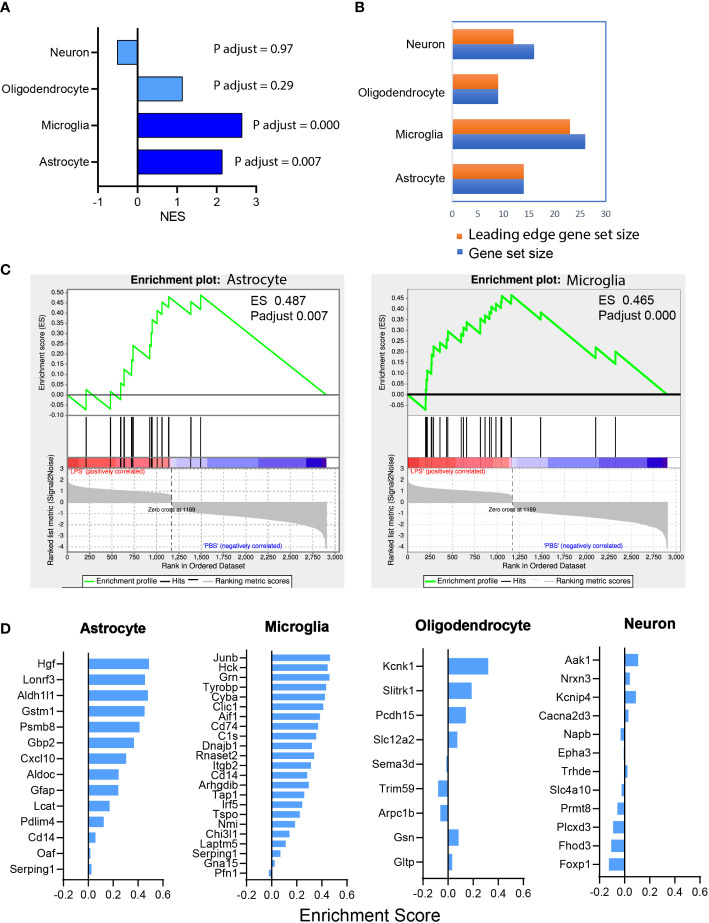
Astrocyte and microglia cell type-specific enrichment was identified in the ONH transcriptome. **(A)** Gene set enrichment analysis (GSEA) was performed based on the differential expression gene list (sorting criteria: P adjust < 0.05, Fold change ≥ 1.0). The normalized enrichment scores (NES) of reference-based cell type-specific gene sets in LPS vs PBS transcriptome are displayed. Astrocytic and microglial gene sets were significantly enriched (P adjust < 0.001) and matched genes showed upregulation in LPS group versus PBS group. **(B)** Counts of the gene sets that were matched in enrichment analysis. Orange, leading edge gene sets; blue, matched gene sets. **(C)** GSEA enrichment plots of astrocyte (ES 0.487, P adjust 0.007) and microglia (ES 0.465, P adjust 0.000) gene sets are shown. **(D)** The leading-edge gene sets for each cell type with the respective ES values.

GSEA of the RNAseq dataset showed that LPS-induced transcriptomic alteration was significantly enriched with astrocyte and microglia cell type-specific gene sets. The normalized enrichment score (NES), a measure of the magnitude of enrichment, was 2.14 for astrocytic gene sets (P < 0.01) and 2.64 for microglial gene sets (P < 0.001), whereas the NES was non-significant for neuron and oligodendrocyte gene sets (**Figure 6A**). GSEA also can be used to identify the leading-edge genes that most strongly contribute to the detected enrichment signal of a pathway (24). We identified 14 of the leading-edge genes for astrocyte, 23 for microglia, 9 for oligodendrocyte, and 12 for neuron from cell type-specific gene sets (**Figures 6B, D**). GSEA enrichment plots for astrocyte and microglia cell types demonstrated that the leading-edge genes mostly showed significant upregulation in the LPS versus PBS groups (**Figure 6C**). In summary, vitreous LPS insults induced ONH transcriptomic alterations that were significantly enriched in astrocyte and microglia cell type-specific gene sets. These findings suggest that vitreous LPS insults predominantly induce glial activation (astrocyte and microglia) at the molecular levels in the ONH.

## Discussion

In the present study, we found that vitreous LPS insults induced glial activation and upregulation of pro-inflammatory cytokines in the retina. In the posterior ON, increased G-ratio of RGC axons and axonal swelling were observed under transmission electron microscopy. In the ONH, fibrous astrocytes flipped the morphological orientation and extended their cellular processes along the longitudinal axis (RGC axonal axis) in vitreous LPS-treated eyes compared with PBS-treated eyes. In the ONH, no obvious alterations were identified in microglial morphology or infiltration of inflammatory cells. However, the RNA sequencing of ONH extracted on day 1 PII revealed striking transcriptomic alterations that were predominantly enriched in astrocyte and microglia cell type-specific gene sets. The current study provides, for the first time, evidences of the morphological and molecular alterations in the rodent ONH in response to acute vitreous LPS insults.

According to the previous literature, axonal transportation can pass vitreous substances into the ON and cerebrospinal fluid circulation (31). The ONH resides between the retina and the ON, with RGC axons and vessels passing through this segment (16, 32, 33). LPS is known to potently induce astrogliosis and microgliosis in the neuroretina (34). Moreover, LPS impairs the blood brain barrier (BBB) and the blood retina barrier (BRB), releasing toxic substances and resulting in secondary cellular injury (35). In theory, the vitreous inflammatory substances can be either transported *via* RGC axons or leak through the impaired BRB. However, compared with the evident neuroinflammation in the retina, we found no signs of glial activation or upregulation of proinflammatory cytokines in the posterior ON after vitreous LPS treatment. Moreover, we found no evidence of inflammatory cell infiltrations from the vitreous cavity into the ONH. These results suggest that the ONH could participate in intrinsic protective mechanisms to prevent external hazards from penetrating into the central nervous system.

**Figure 7:**
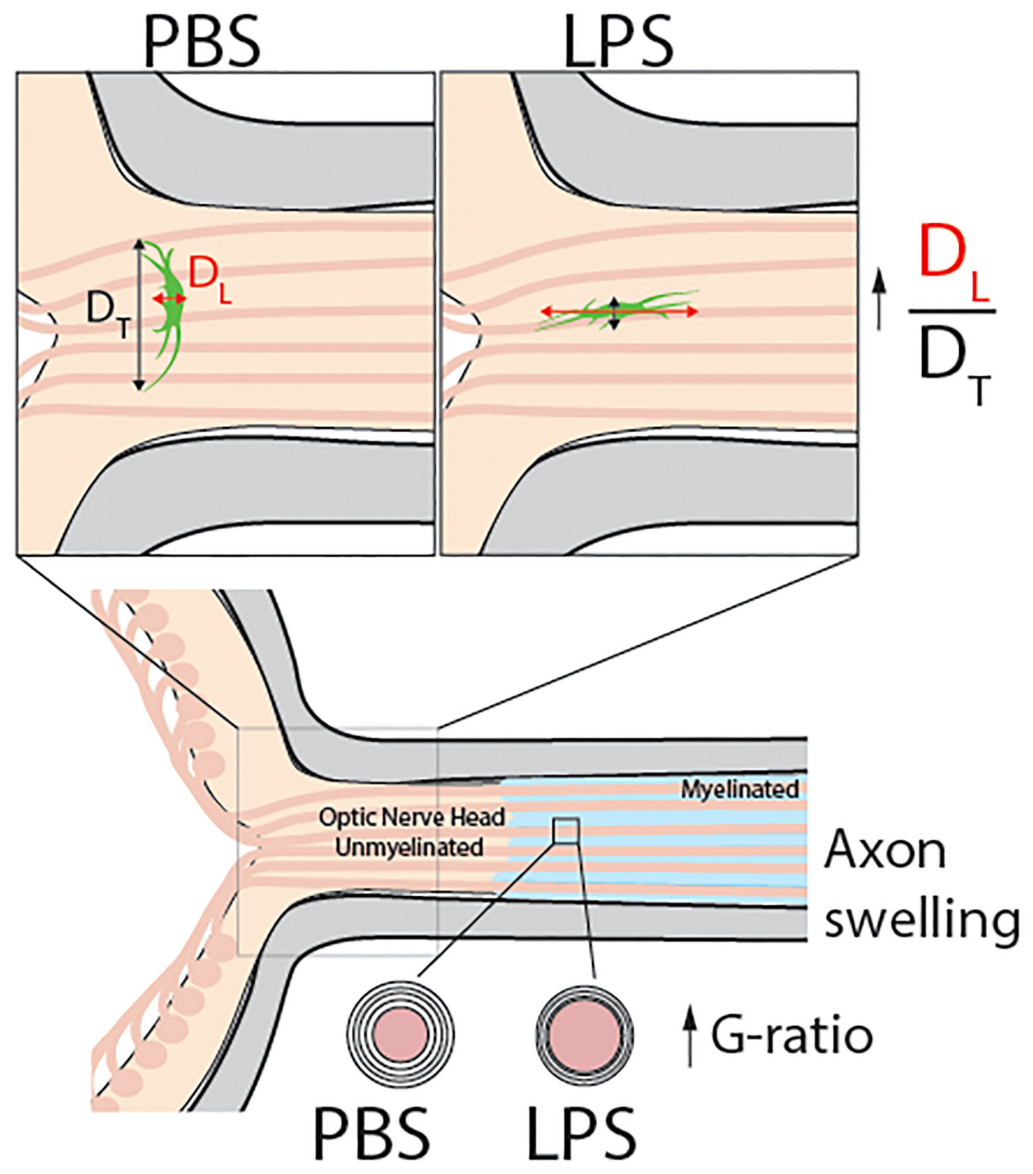
Model abstract. This study reports that vitreous infectious insults cause evident neuroinflammation in the retina but not in the posterior ON with no infiltrating inflammatory cells observed in the ONH on day 1 PII. Using transmission electron microscopy, we observed mild but significant demyelination in the posterior ON. Confocal imaging revealed a dramatic morphological transformation of ONH astrocytes, which was quantified by the increase the ratio of longitudinal (DL) and transverse (DT) diameters of ONH fibrous astrocytes compared to PBS-treated eyes. These results show that in response to vitreous infectious insults, the ONH astrocytes alter their spatial orientation by elongating their morphology along the axonal axis of RGCs. Along with ONH transcriptomic data, we believe vitreous LPS insult- induced glial activation in the ONH serves as a barrier preventing vitreous inflammation from penetrating into the central nervous system.

The cellular components of the ONH, termed glial lamina in rodents and lamina cribrosa in human, have been found in prevailing literature (14, 36–38). In human eyes, laminar beams can be imaged as a shelf, with fibrous astrocytes and lamina cribrosa cells growing along with them. The ECM fills the space and builds a “water-proof wall” (38). In the glial lamina of rodent models, RGC axons are directly ensheathed by fibrous astrocytes instead of oligodendrocyte-derived myelin (39)and also shown in the images generated by TEM (**Figure 4E**). Calkins *et al.* reported that the direct interface of fibrous astrocytes and RGC axons provides the anatomical basis for cross-cellular metabolism (40). Other research has found that the rodent ONH fibrous astrocytes degrade the mitochondria in RGC axons, supporting the hypothesis that the ONH fibrous astrocytes can directly regulate the RGC physiological or pathological processes (41). The most striking data were the morphological transformation of ONH fibrous astrocytes (**Figure 4**). We found that vitreous acute inflammation can flip the morphological ratio (D_T_/D_L_) of ONH astrocytes, suggesting changes in the spatial colocalization of ONH astrocytes and the RGC axons. Astrocytes extend their cellular processes along the longitudinal axis (RGC axonal axis), which might result in a loose contact with unmyelinated axons and leave more space in the glial lamina (**Figure 7**). Numerous studies reported the reactive astrocytes could be either beneficial or detrimental to nerve tissue in CNS insult (42). Subtypes of astrocytes have been categorized by molecular expressions and reactivity towards insults (43). Excessive activation of cortical protoplasmic astrocytes loss isolated cell territories and induce seizures (44). Fibrous astrocytes of white matter interdigitate the neighboring astrocytes with processes and undergo structural remodeling in formation of glial scar that could block the neuronal regeneration (29). Based on our observation, we speculate that ONH astrocyte could reduce the ensheathing of the unmyelinated RGC axons, interfere with the axonal metabolisms, and possibly dampen the barrier function in the later time points. The role of astrocyte might vary depending on the severity or duration of insults and the time points post injury. Evidences from models of glaucoma have shown that chronic intraocular pressure is able to re-organize the ECM and alter fibrous astrocytic morphology (15, 20, 45, 46). Similar astrocytic morphological changes as we observed here was also reported in the model of glaucoma (17). It was recently reported the fluid transportation barrier of ONH was impaired in glaucoma mice (31). However, the interactions between neuroinflammation and pressure-induced chronic stress in the pathogenesis of glaucoma remain unclear (47).

Numerous studies have found that microglia are highly sensitive to the surrounding microenvironment and perform active morphological dynamic changes with the presence of LPS (48). It also has been proposed that neurotoxic reactive astrocytes are induced by activated microglia through the release of inflammatory stimulating substances (49). Based on GSEA, we found evidences that both ONH astrocytes and microglia were drastically activated by LPS intravitreal injection. However, we did not observe the microglial morphology alterations in the ONH on day 1 PII. The different result from RNAseq and morphological analysis could be due to the limitation of morphological analysis in detecting the microglial activation in the tissue of ONH. The absence of observable microglia activation after single dosage of LPS intraperitoneal injection were reported in previous studies, the activated microglia was then detected after multiple administration of LPS (50, 51). Although Sholl analysis showed unaltered microglia morphology following LPS administration, GSEA result indicated activation of microglia at mRNA level. Further studies are required to explore the functional roles of glial cell activation in this process. However, the available evidence supports the hypothesis that ONH glial cell activation in response to acute vitreous infectious insults can prevent the penetration of vitreous inflammation into the ON.

## Conclusion

In this study, we provided experimental evidence that in rodent models, vitreous infectious insults induced morphological and molecular alterations of glial cells in the ONH in the acute phase. The ONH is likely to function as a barrier preventing vitreous neuroinflammation from penetrating into the central nervous system. It remains unclear whether ONH glial activation is protective or damaging to RGC axonal injuries. Targeting ONH glial cells may have translational potential to relieve RGC retroocular degeneration or injuries.

## Data availability statement

The datasets presented in this study can be found in online repositories. The names of the repository/repositories and accession number(s) can be found below: NCBI (SRA) under accession ID PRJNA796494.

## Ethics statement

The animal study was reviewed and approved by The Institutional Animal Care and Use Committee of Tongji Hospital, Tongji Medical College, Huazhong University of Science and Technology, Shanghai Medical College, Fudan University.

## Author contributions

WL and JH, conducted the experiments and data analysis. SD and YX, contributed in data processing, WL, FD, WF, and XW prepared the figures and manuscripts. TQ and WW commented on the manuscript and provided professional suggestions. FD and XW designed the experiments and sponsor the study. All authors contributed to the article and approved the submitted version.

## Funding

This work was supported by the National Key R&D Program of China, 2021YFC2502200, the Clinical Research Plan of SHDC (SHDC2020CR2041B), the National Nature Science Foundation of China [grant numbers 8202010801, 32070984, 81801318], and Shanghai Scientific Society, General Project [grant number 20ZR1403500, 21QA1401100].

## Acknowledgments

We are grateful that Dr. Song Qin generously provided the mouse strain of Aldh1L1-eGFP. We thank for Dr. Zhili Huang for inspiring suggestions for experiment design. We appreciate Dr. Chenju Yi for valuable comments for the manuscript and figures.

## Conflict of interest

The authors declare that the research was conducted in the absence of any commercial or financial relationships that could be construed as a potential conflict of interest.

## Publisher’s note

All claims expressed in this article are solely those of the authors and do not necessarily represent those of their affiliated organizations, or those of the publisher, the editors and the reviewers. Any product that may be evaluated in this article, or claim that may be made by its manufacturer, is not guaranteed or endorsed by the publisher.

## Supplementary material

Supplementary Figure 1 Measurements of retinal thickness on day 1 and 7 PII of PBS and LPS **(A)** Schematic illustration of tissue section preparation and the image fields selected in the retina. Six images of 20 x magnification were taken at position 1-6 on the retina. Considering the thickness might vary due to the positions, we grouped the symmetric positions (1 and 6, 4 and 5, 3 and 4) and compared between LPS and PBS groups on PII 1d or PII 7d respectively. **(B)** Representative image of the thickness measurements. **(C, D)** Comparisons of retinal thickness at position 1 and 6, 4 and 5, 3 and 4 between the PBS and LPS groups on day 1 and 7 PII. For each pair of comparison, the retinal thickness was normalized to the average of the PBS group measurements. Data was presented as the mean ± SD. Unpaired t-test was performed for statistical analysis, ns, non-significant.

Supplementary Figure 2 fMOST imaging of ONH astrocytes **(A)** Schematic illustration of experiment using the technique of fMOST in the fixed tissue sample of optic nerve head (ONH) generated from Aldh1L1-eGFP reporter mice. **(B)** The demonstration of the spatial location of ONH in 3D-construct of the entire tissue sample (left, scale bar, 1000 μm), 2D-sagittal section (middle, scale bar, 300 μm), 3D-construct of ONH (right, scale bar,100 μm). Outlined planes on the right panel indicate the locations for 2 representative coronal sections displayed in panel G. n = 1 ON for each group from one mouse. **(C)** Representative images of coronal sections extracted from ONH for both groups. scale bar, 40 μm, **(D)** Representative images of coronal sections extracted from ONH for both groups, scale bar, 30 μm.

Supplementary Figure 3 The top 20 up and down-regulated differentially expressed genes **(A)** Cluster heatmap demonstrating the transcriptomic features of top 20 up-regulated differentially expressed genes (DEGs) in LPS group in comparison with PBS group. Differential expression analysis was performed using the statistical DESeq2 (p-adjust ≤0.01 and | log2Fold Change | ≥ 1). The genes were ranked based on the fold change. **(B)** Cluster heatmap demonstrating the transcriptomic features of top 20 downregulated DEGs in LPS group in comparison with PBS group.
