# Supplementary figures and images for: Optic nerve head: A gatekeeper for vitreous infectious insults?
Source: Front Immunol. 2022 Sep 20;13:987771. doi: 10.3389/fimmu.2022.987771 (PMC9531234; doi:10.3389/fimmu.2022.987771)

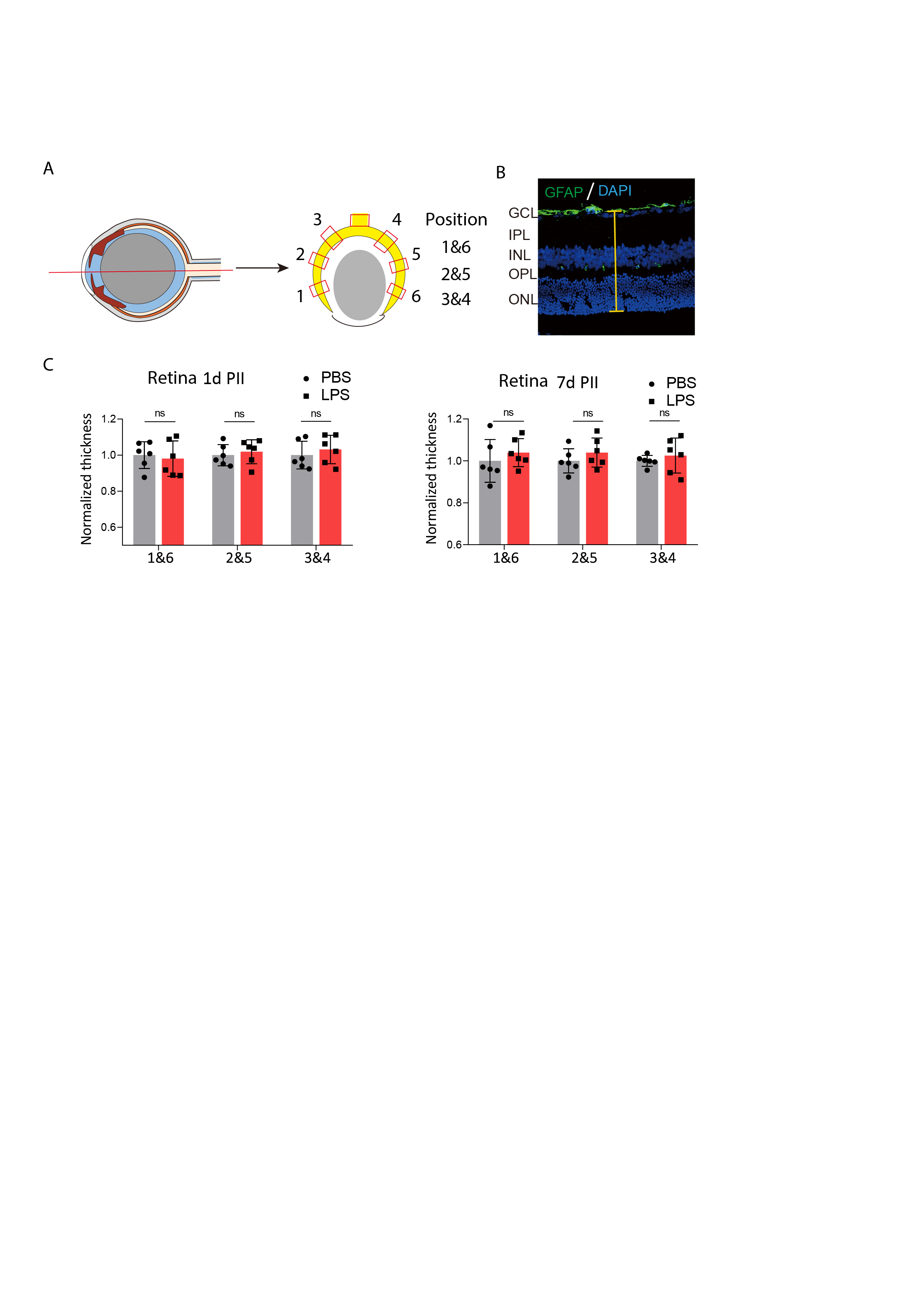

Supplement: Supplementary Figure 1 — Measurements of retinal thickness on day 1 and 7 PII of PBS and LPS (A) Schematic illustration of tissue section preparation and the image fields selected in the retina. Six images of 20 x magnification were taken at position 1-6 on the retina. Considering the thickness might vary due to the positions, we grouped the symmetric positions (1 and 6, 4 and 5, 3 and 4) and compared between LPS and PBS groups on PII 1d or PII 7d respectively. (B) Representative image of the thickness measurements. (C, D) Comparisons of retinal thickness at position 1 and 6, 4 and 5, 3 and 4 between the PBS and LPS groups on day 1 and 7 PII. For each pair of comparison, the retinal thickness was normalized to the average of the PBS group measurements. Data was presented as the mean ± SD. Unpaired t-test was performed for statistical analysis, ns, non-significant. [file Image_1.tif]

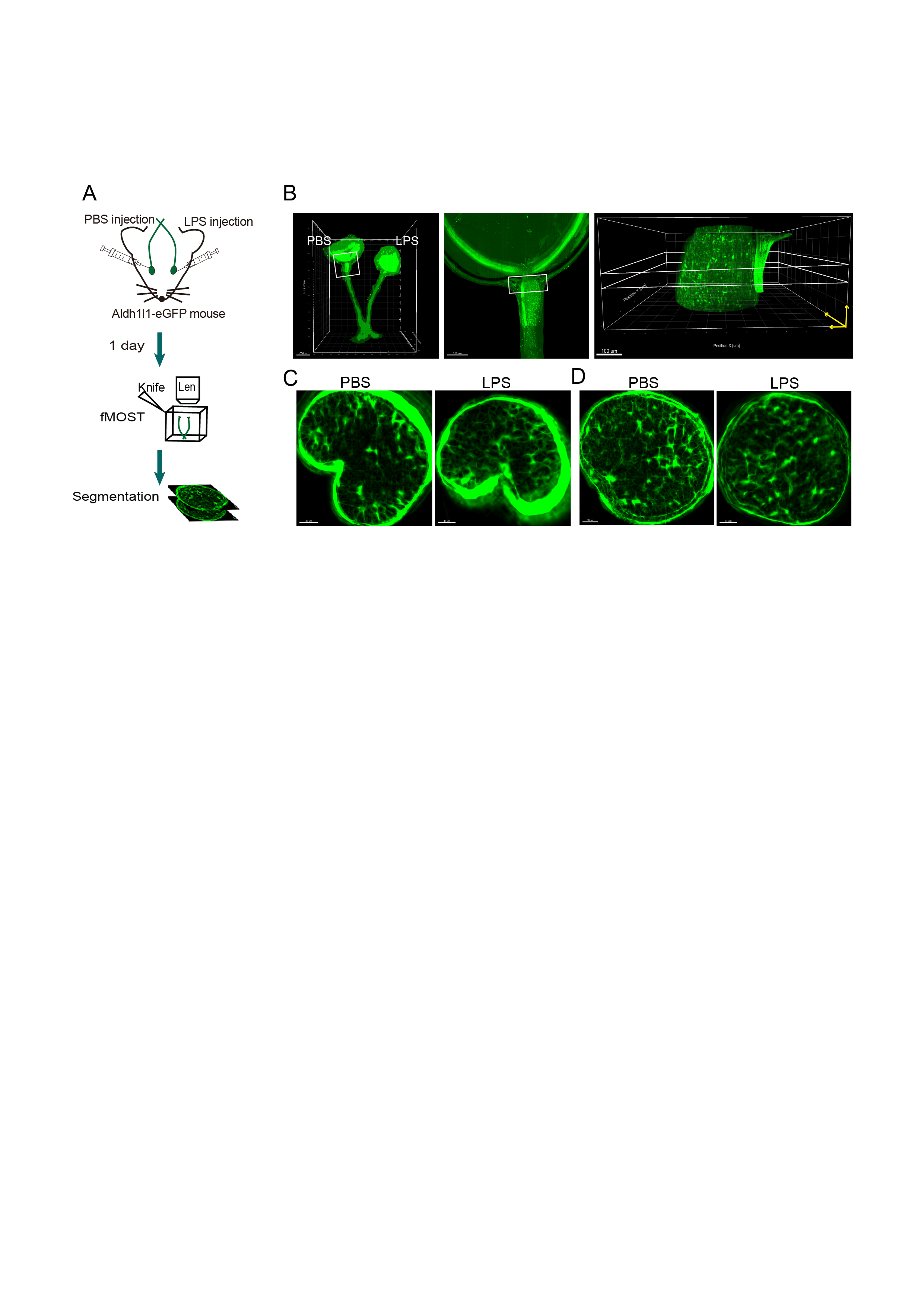

Supplement: Supplementary Figure 2 — fMOST imaging of ONH astrocytes (A) Schematic illustration of experiment using the technique of fMOST in the fixed tissue sample of optic nerve head (ONH) generated from Aldh1L1-eGFP reporter mice. (B) The demonstration of the spatial location of ONH in 3D-construct of the entire tissue sample (left, scale bar, 1000 μm), 2D-sagittal section (middle, scale bar, 300 μm), 3D-construct of ONH (right, scale bar,100 μm). Outlined planes on the right panel indicate the locations for 2 representative coronal sections displayed in panel G. n = 1 ON for each group from one mouse. (C) Representative images of coronal sections extracted from ONH for both groups. scale bar, 40 μm, (D) Representative images of coronal sections extracted from ONH for both groups, scale bar, 30 μm. [file Image_2.tif]

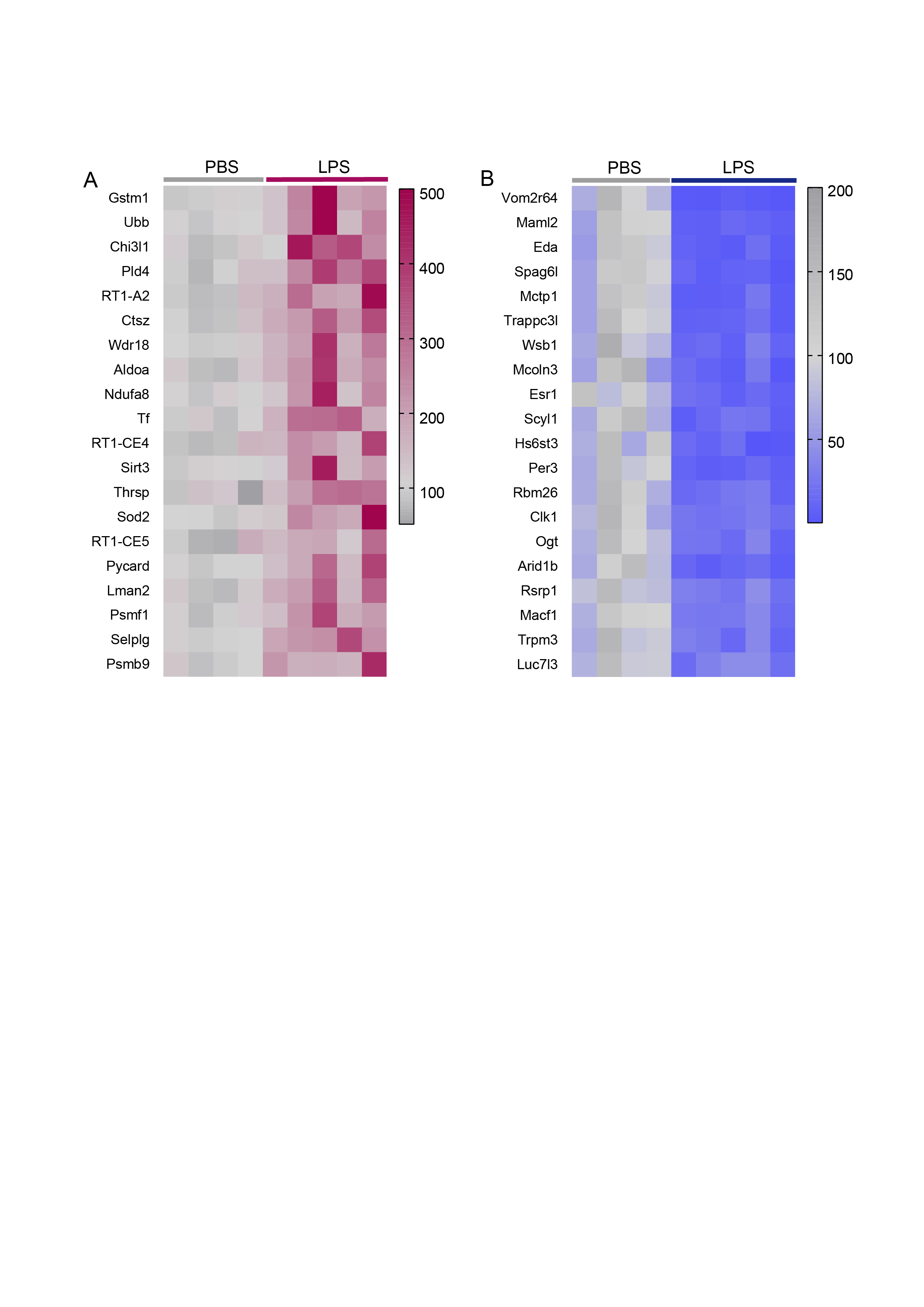

Supplement: Supplementary Figure 3 — The top 20 up and down-regulated differentially expressed genes (A) Cluster heatmap demonstrating the transcriptomic features of top 20 up-regulated differentially expressed genes (DEGs) in LPS group in comparison with PBS group. Differential expression analysis was performed using the statistical DESeq2 (p-adjust ≤0.01 and | log2Fold Change | ≥ 1). The genes were ranked based on the fold change. (B) Cluster heatmap demonstrating the transcriptomic features of top 20 downregulated DEGs in LPS group in comparison with PBS group. [file Image_3.tif]
